# Mental health professional impact of dualistic approach on the biological model of depression

**DOI:** 10.1192/j.eurpsy.2025.1292

**Published:** 2025-08-26

**Authors:** Ž. Kralj, S. Melissa Pranić, T. Mastelić, T. Borovina Marasović, T. Kozina, S. Kozina

**Affiliations:** 1 Department of Psychiatry, University Hospital of Split; 2 Department of Psychiatry; 3 Department of Public Health, School of Medicine University of Split; 4 Department of Professional Studies, University of Split; 5 Department of Psychological Medicine, School of Medicine University of Split, Split, Croatia

## Abstract

**Introduction:**

In psychopathology, a dualistic approach often refers to the conceptual separation of the mind and body as distinct entities, which can influence how mental disorders are classified and diagnosed. This separation may lead to biased judgments and contribute to the stigmatization of mental health conditions. It can also encourage alternative conceptualizations of mental health disorders and promote new methods of classifying mental health issues beyond traditional biomedical or behavioral frameworks.

**Objectives:**

The goal of our research was to examine the impact of psychiatrists’ and psychologists’ dualistic perspectives on the biological model of depression using the PLS-SEM model.

**Methods:**

This cross-sectional study carried out in the Republic of Croatia in 2018, involved 238 participants, comprising 122 psychiatrists and 116 psychologists. We applied the Maudsley Attitude Questionnaire to examine the preferences of psychiatrists and psychologists in choosing the biological model of depression. Their dualistic perspectives were assessed using the Stanovich Mind-Body Dualism Scale. In the PLS-SEM modeling, dualism and monism were observed as exogenous latent variables in the model, and their impact on the biological model, which represents the endogenous latent variable, was analyzed. All model indicators are reflective.

**Results:**

Psychiatrists (M = 14.71, SD = 2.27) and psychologists (M = 13.77, SD = 2.69) predominantly support the biological model out of Harlands’ 8 models of mental disorders in defining depression. The PLS-SEM initial reflective model is significant (p = 0.002) even though the fit indices provided mixed results. The GFI (0.963) and SRMR (0.059) suggested a good or acceptable fit, but the CFI (0.862) and RMSEA (0.095) indicated that the model requires further refinement. The R² value revealed that the model explained 61.3% of the variance in the endogenous variables.

**Conclusions:**

Mental health professionals prefer the biological approach in explaining the etiology, classification, research, and treatment of depression. Supporting a dualistic perspective had a significant negative total effect (p < 0.05) on the choice of the biological model of depression.

**Disclosure of Interest:**

None Declared

